# Doctors and the Bill

**Published:** 1911-06-10

**Authors:** 


### Doctors and the Bill.

During the past week attempts have been made to show that the medical profession is wholly against the Insurance Bill. A glance at the composition of the various protesting societies will be sufficient to show the unprejudiced critic that the resolutions adopted as the result of the discussion of the Bill are by no means the unanimous expression of opinion from the profession. Nevertheless we think that the discussion, premature as the resolutions are, and marked as some of the speeches of the protestants have been by a profound ignorance of the essentials of State Insurance and the doctors' condition in other countries, has ventilated the subject of the relation of the doctor to the scheme in a fashion that will direct the attention of the Government and the general public to some outstanding defects of the Bill.

Mr. George has been generous in his explanations to the profession. With his explanations most of us will be content, for it is a lamentable fact that the difficulties discussed have arisen out of a prejudiced interpretation of some of the clauses of the Bill, and from a desire, implied if not expressed, to make communal subservient to class interest. The State cannot allow itself to be dictated to by a trade-union in a matter which affects the general welfare of the community, and the sooner the profession realises this the better for our dignity and reputation. The objection to the wage-limit, made so much of during the past week, is a foolish and prejudicial objection which, if persisted in, will confirm the public more than ever in its conception of the medical profession in this country as a huge technical trust which, in Sir William Collins' words, is "out for fees." The difficulties, presumably, which aghast some of us in this connection may be overcome by the adoption of a sliding scale of contributions, as laid down in the latest novello to the German Act, whereby the proportionate fees paid for medical attendance vary in the different classes. It is quite true the German scheme limits the wage to £100 per year in the case of the compulsory insured, but it allows those in possession of an income above that limit to join the insurance societies and to benefit under the Act. The limit fixed by the Bill is, therefore, in our opinion, a reasonable and modest one which we trust will be adhered to, for we are convinced that members of the medical profession will speedily see that the introduction of State Insurance tends to promote their best interest as a profession, though it tends, equally, to militate against their interests as a trade.

In this matter it is necessary that the general practitioner, who has a thorough experience of club practice, should express an opinion. It is foolish to let the specialist or consultant, who is absolutely without practical experience of club and private work, be his mouthpiece and voice his sentiments, often in a manner which is prejudiced so obviously that the public may be pardoned for refusing to accept the opinions as those of serious, practical men. Here again we would commend to our readers a close study of the Bill and of the models on which it is founded. A consideration of the professional status of the German general practitioners with that of our own practitioners will show that State Insurance across the Channel has not tended to lower but rather to increase the prestige of doctors, and to extend the usefulness of the profession to the public.

